# Thyroid disorders in psychiatric patients: a descriptive study in a psychiatric hospital

**DOI:** 10.1192/j.eurpsy.2023.1008

**Published:** 2023-07-19

**Authors:** U. López, L. Morado San Segundo, C. González Navarro, I. Alonso Salas, A. López Fariña, A. Bilbao Idarraga, B. Samsó Martínez, R. F. López Brokate, E. M. Garnica de Cos, T. Ruiz de Azua Aspizua, U. Ortega Pozas

**Affiliations:** 1 Red de Salud Mental de Bizkaia, Bilbao, Spain

## Abstract

**Introduction:**

Thyroid disorders can present with psychiatric symptons similar to depression, and, at the same time, certain treatments, like litio, can cause changes in thyroid function. Given, therefore, the importance for the treatment and care of patients, the study of thyroid function is one of the parametres that should be requested in patients with psychiatric pathology.

**Objectives:**

To study the frequency of thyroid disorders in patients who where admitted to a psychiatric short stay unit.

**Methods:**

Retrospective descriptive observational study is carried out in the acute stay unit of a psychiatric hospital. As a sample, all patients admitted to the unit over a period of three months. During admission, their sociodemographic data, the treatment they receive and their diagnosis are recorded. Secondly, blood test are performed whith differents parameters, including TSH values.

**Results:**

In the total sample of 172 patients, 8 of them have TSH abnormalities. 7 of them, all women, present hypothyroidism values.

A single male patient presented values of hyperthryroidism.

**Conclusions:**

According to the present study, 4,6% of the patients present alterations at the TSH at admission, although except in one case, the values were not markedly altered.

The thyroid study at admission allows detecting cases of altered TSH that are amenable to treatment and monitoring.

**Disclosure of Interest:**

None Declared

